# Flavokawain B targets protein neddylation for enhancing the anti-prostate cancer effect of Bortezomib *via* Skp2 degradation

**DOI:** 10.1186/s12964-019-0338-2

**Published:** 2019-03-18

**Authors:** Xuesen Li, Victor Pham, Matthew Tippin, Dongjun Fu, Raymond Rendon, Liankun Song, Edward Uchio, Bang H. Hoang, Xiaolin Zi

**Affiliations:** 1 Department of Urology, University of California, Irvine, 101 The City Drive South, Rt.81, Bldg.55, Rm.302, Irvine, Orange, CA 92868 USA; 2 Institute for Cancer Medicine and School of Basic Medical Sciences, Southwest Medical University, Luzhou, Sichuan 646000 China; 3 Pharmaceutical Science, University of California, Irvine, Orange, CA 92868 USA

**Keywords:** Chalcone, Neddylation, Skp2, And prostate cancer

## Abstract

**Background:**

Flavokawain B (FKB) has been identified from kava root extracts as a potent apoptosis inducer for inhibiting the growth of various cancer cell lines, including prostate cancer. However, the molecular targets of FKB in prostate cancer cells remain unknown.

**Methods:**

An in vitro NEDD8 Initiation Conjugation Assay was used to evaluate the neddylation inhibitory activity of FKB. Molecular docking and a cellular thermal shift assay were performed to assess the direct interaction between FKB and the NEDD8 activating enzyme (NAE) complex. Protein neddylation, ubiqutination, stability and expression in cells were assessed with immunoprecipitation and Western blotting methods using specific antibodies. Deletion and site specific mutants and siRNAs were used to evaluate deep mechanisms by which FKB induces Skp2 degradation. Cell growth inhibition and apoptosis induction were measured by MTT, ELISA and Western blotting methods.

**Results:**

FKB inhibits NEDD8 conjugations to both Cullin1 and Ubc12 in prostate cancer cell lines and Ubc12 neddylation in an in vitro assay. Molecular docking study and a cellular thermal shift assay reveal that FKB interacts with the regulatory subunit (i.e. APP-BP1) of the NAE. In addition, FKB causes Skp2 degradation in an ubiquitin and proteasome dependent manner. Overexpression of dominant-negative cullin1 (1–452), K720R mutant (the neddylation site) Cullin1 or the F-box deleted Skp2 that losses its binding to the Skp1/Cullin1 complex causes the resistance to FKB-induced Skp2 degradation, whereas siRNA knock-down of Cdh1, a known E3 ligase of Skp2 for targeted degradation, didn’t attenuate the effect of FKB on Skp2 degradation. These results suggest that degradation of Skp2 by FKB is involved in a functional Cullin1. Furthermore, proteasome inhibitors Bortezomib and MG132 transcriptionally down-regulate the expression of Skp2, and their combinations with FKB result in enhanced inhibitory effects on the growth of prostate cancer cell lines via synergistic down-regulation of Skp2 and up-regulation of p27/Kip1 and p21/WAF1 protein expression. FKB also selectively inhibits the growth of RB deficient cells with high expression of Skp2.

**Conclusion:**

These findings provide a rationale for further investigating combination of FKB and Bortezomib for treatment of RB deficient, castration-resistant prostate cancer.

**Electronic supplementary material:**

The online version of this article (10.1186/s12964-019-0338-2) contains supplementary material, which is available to authorized users.

## Background

Targeted and combined cancer treatments have significantly increased in demand as the side effects and resistant mechanisms of common therapies have been researched in greater detail. Neural Precursor Cell Expressed, Developmentally Down-Regulated 8 (NEDD8), an ubiquitin-like protein, plays an important role in the modification of Cullin-1 to turn on the Skp1-Cullin-F box protein (SCF) complex for regulation of the stability of its target proteins [1]. The neddylation of Cullin1 occurs via a conjugation cascade-the neddylation pathway, which is initiated by an E1 (i.e. NEDD8 activating enzyme, NAE) enzyme consisting of Amyloid Precursor Protein-binding Protein1 (APP-BP1) and Ubiquitin-Like Modifier Activating Enzyme 3 (UBA3) proteins. Activated E1 then transfers NEDD8 to its E2 enzyme NEDD8-conjugating enzyme 2 M (UBE2M), also called Ubc12, which causes covalent modulation of Cullin proteins with NEDD8 for activation of Cullin-RING ubiquitin ligases. Many components of the neddylation pathway, such as NEDD8, NAE and DCN1, have been reported to be over-expressed in several cancers [2–4]. In addition, high levels of NEDD8 mRNA were related to resistance to Bortezomib in multiple myeloma patients [5]. Therefore, the neddylation pathway could be targeted for development of novel cancer therapies. Indeed, a small molecule inhibitor of NAE, MLN4924 (a first-in-class inhibitor of NAE also named as pevonedistat), has been developed and currently in multiple phase I/II clinical trials for patients with advanced solid tumors or hematological tumors [6–10]. However, results from initial trials suggested that MLN4924 as a single agent has limited anti-tumor efficacy and is dose limiting because of toxicities. Therefore, there is a need for development of more efficient or less toxic NAE inhibitors or novel combination therapies.

Natural products have long been a rich resource for identifying novel anti-cancer agents with relatively few side effects. Flavokawain B (FKB) is a naturally occurring chalone identified in the Kava plant. FKB has been shown potent anti-tumor activities in xenograft models of a variety of cancers, including in human gastric carcinoma, breast and prostate cancers in nude mice [11–17]. We have demonstrated that FKB selectively inhibited the growth of androgen receptor negative, castration resistant prostate cancer cell lines with minimal effects on the growth of normal prostate epithelial and stroma cells [13]. We and other researchers have observed that the cancer specific cytotoxicity of FKB is associated with the generation of intracellular reactive oxygen species and up-regulation of death receptor-5 and Bim expression, which leads to induction of G2M arrest and apoptosis [13, 15, 18]. However, the molecular targets of FKB in cancer cells remain unclear. In this study, we have shown that FKB inhibits NEDD8 conjugations to both Cullin1 and Ubc12 in prostate cancer cell lines and Ubc12 NEDDylation in an in vitro assay. Molecular docking study and a cellular thermal shift assay (CETSA) has further indicated that FKB directly interacts with the regulatory subunit *(*i.e. APP-BP1) of the NAE. These results together suggest that FKB is a novel NAE inhibitor.

The neddylation status of the SCF complex is essential for its function on degradation of both its substrate and itself [19]. Moreover, individual components of the SCF complex, including Cullin1, S-phase kinase associated protein 2 (Skp2), copper metabolism domain containing 1 (Commd1), and cyclin-dependent kinases regulatory subunit 1(Cks1b) have been linked to Bortezomib resistance in multiple myeloma [20]. Here, we have observed that FKB induces a proteasome-dependent and ubiquitin-mediated degradation of Skp2 and that the effect of FKB on Skp2 degradation relies on a functional Cullin1. We have further shown that FKB markedly enhances the growth inhibitory and apoptotic effect of proteasome inhibitors MG132 and Bortezomib via down-regulation of Skp2 and upregulation of p27/Kip1 and p21/WAF1 protein expression. In addition, FKB preferentially inhibits the growth of RB deficient cells compared to RB wild-type cells. Our study suggests that FKB and Bortezomib combination deserves further investigation for treatment of RB deficient late stage prostate cancer.

## Materials and methods

### Cell culture

The prostate cancer cell lines LNCaP and PC3 were obtained from American Type Culture Collection (ATCC, Manassas, VA), while C4-2B prostate cancer cell lines were purchased from Urocor Inc. (Oklahoma City, OK). These cells were characterized and authenticated by ATCC or Urocor Inc. In addition, all cell lines were tested for known species of mycoplasma contamination using a kit from LONZA Inc. (Walkersville, MD). These prostate cancer cells were cultured in RPMI-1640 media (Fisher Scientific) supplemented with 10% fetal bovine serum (FBS), 1% L-Glutamine, and 1% penicillin-streptomycin as described previously in our publication [12, 13]. RB +/+ and RB −/− mouse embryonic fibroblasts (MEFs) and mouse prostate epithelial cells (MPECs) were obtained from Dr. Wen-Hwa Lee at the University of California, Irvine and from Dr. Scott D Cramer at Wake Forest University School of Medicine, respectively. These cells were grown in Dulbecco’s modified Eagle’s media (DMEM) that has supplements of 10% FBS, 1% L-Glutamine, and 1% Penicillin-Streptomycin. All cell lines used in this study at passage 15–20 were used for all experiments.

### Compounds, antibodies, and reagents

FKB with 99% purity was isolated from kava extracts by LKT Laboratories, Inc. (St. Paul, MN). Bortezomib, MG-132 and MLN4924 were obtained from Cayman Chemical Inc. (Ann Arbor, MI). Antibodies against Ubc12, ubiquitin, and β-tubulin were from Santa Cruz Biotechnology, Inc. (Santa Cruz, CA). Anti-Skp2 antibodies were purchased from Invitrogen (Grand Island, NY). Anti-Myc-tag and Anti-cleaved-PARP antibodies were from Cell Signaling (Boston, MA). Anti-Cullin-1 and anti-NEDD8 antibodies were from Abcam (Cambridge, MA). Anti-p27/Kip1 and p21/WAF1 antibodies were from BD Biosciences (Billerica, MA). 3-(4, 5-dimethylthiazol-2-yl)-2, 5-diphenyltetrazolium bromide (MTT) was purchased from Sigma. The Reverse Transcription System kit and was from Promega (Mandison, WI). A quantitative reverse transcription polymerase chain reaction (RT-PCR) kit was from Bio-Rad (Hercules, CA). Ubiquitylation Assay Kit and 20S Proteasome Assay Kit were from Cayman and Abcam, respectively.

### MTT assay

Cells (2 × 10^4^ cells/well) were grown in 24-well culture dishes for 24 h, and then treated as indicated in the figures. Cells were then incubated for another 48 h before adding 1 mg/mL MTT in 20% PBS and 80% culture medium (*v*/v) for 2 h. The absorbance was read at 570 nm, and the dose-response curves for reduction of cell viability were generated as percentage ratios of vehicle-treated controls.

### Western blot analysis

Cellular protein lysates (20-100μg) were denatured in 2X loading buffer at 100 °C prior to adding into 8–16% SDS-PAGE. For non-denatured Western blotting analysis, non-reducing loading buffer were mixed with protein lysates prior to resolving it in non-denaturing gel (Biorad, CA). Proteins were transferred to nitrocellulose membranes, probed with indicated antibodies, and visualized by an enhanced chemiluminescence detection system. The western blotting bands were semi-quantified using Image J and adjusted for loading controls, β-actin or tubulin.

### Plasmid and siRNA transfection

Plasmids of PcDNA-Skp2/myc and pGL2-Skp2 promoter-luciferases (Skp2-Luc) were from Addgene No. 19947 and No. 81119, respectively [21]. PcDNA-Skp2/myc and control vector PCDNA were transfected into PC3 cells with Fugene 6 from Roche (Indianapolis, IN), and stable clones were screened for positive expression of Skp2 and mixed positive clones used in the experiments. Delta-B box Cullin-1 (dominant-negative Cullin-1 (1–456)) plasmid was a kindly gift from Dr. Zhen-Qiang Pan (Derald H. Ruttenberg Cancer Center, New York) [22]; delet-F-box-Skp2-V5 was a kindly gift from Dr. Thilo Hagen (National University of Singapore) [23]. All the siRNAs, including siSkp1, siCSN5, siUbc12, and siCdh1, were from Qiagene (Valencia, CA). All the transient transfections were performed using Lipofectamin 2000 from Invitrogen.

### Promoter activity and luciferase assay

PC3 cells were co-transfected with Skp2-Luc and Renilla luciferase plasmid pGL 4.71 (Promega) by Lipofectamine 2000 (Invitrogen). After 48 h of transfection, FKB was added as indicated with triple replications. Then cells were harvested and luciferase activity was measured with the Dual-Glo Luiferase assay system (Promega). Renilla luminescence was used as an inner control for cell numbers and transfection efficiency. The relative ratio of luminescence from interested gene promoter to Renilla luminescence was shown in the figures as promoter activity.

### In vitro NEDD8 initiation conjugation assay

The NEDD8 initiation conjugation assay kit was purchased from Bonston Biochem (Cambridge, MA). A master mix of 0.4 μM APP-BP1/UBA3 (NEDD8 ligase E1), 12.5 μM UbcH12 (NEDD8 ligase E2) and 62.5 μM NEDD8 were prepared in the reaction buffer (pH 8.0, 50 mM HEPES and 50 mM NaCl in final reaction) and distributed to individual tubes with a volume of 15 μl. A series of dilutions of FKB were made in DMSO. One microliter of FKB or DMSO were added to the indicated tubes and mixed well. The reactions were started by adding 2.5 mM Mg2+ and 1 mM ATP (4 μl in mixture), except the negative control tube was added by equal volume of ddH_2_O. The reaction tubes were incubated in 37 °C for 30 min and stopped by adding 5 μl 25 mM EDTA. Non-reducing western blot was performed with anti-UbcH12 antibody to detect both Ubc12 bands and Nedd8 conjugated Ubc12 bands.

### Cellular thermal shift assay [24]

PC3 cells were washed in 1X PBS before splitting evenly at 1 × 10^6^ cells into 2-mL centrifuge tube in complete medium. One of the 2-mL centrifuge tubes were treated with 0.1% DMSO, while others were treated with indicated concentrations of FKB. After 2 h incubation at 37 °C in a water bath, the cells were washed and suspended in 600 μL of 1X cold PBS that containing protease inhibitor. Each of the treated cells were split evenly into nine new 2-mL centrifuge tubes, labeled with temperature ranging from 40 to 62 °C in increments of 3 degrees. The cells were incubated for 3 min at the indicated temperature, followed by 3 min incubation at room temperature before snap-frozen in liquid nitrogen. The cells were then lysed via snap-frozen in liquid nitrogen, thawing at 25 °C, and brief vortexing three times, before being spun down at 13,000 rpm for 15 min at 4 °C. Prior to heating at 75 °C for 10 min, 40 μL of the supernatants were mixed with 10 μL 5X loading dye containing β-mercaptoethanol, and then used for Western Blot analysis. Primary antibodies against the protein of interest were used.

### Cellular isothermal dose response assay [24]

Similarly to the CESTA experiment, PC3 cells were washed in 1X PBS and split evenly at 1 × 10^6^ cells into eight 2-mL centrifuge tubes. The eight sets of 2-mL centrifuge tubes were treated with increasing concentrations of FKB as shown in the Figure legend, leaving one 2-mL centrifuge tube with 0.1% DMSO as a vehicle control. After 2 h of incubation at 37 °C, the cells were washed with 1X PBS and suspended in 40 μL of PBS containing protease inhibitor. The cells were incubated at an appropriate temperature for 3 min and left at room temperature for 3 min before snap frozen in liquid nitrogen. Cells were lysed and Western blotting analysis was performed as described in the CESTA method.

### Statistical analysis

Comparisons of cell viabilities, fold change in levels of mRNA, caspase activities and ubiquitination and protein levels between different treatments were conducted using Student’s t-test. All statistical tests were two sided. *P* < 0.05 was considered statistically significant.

## Results

### FKB inhibits Cullin-1 and Ubc12 neddylation

**Fig. 1:**
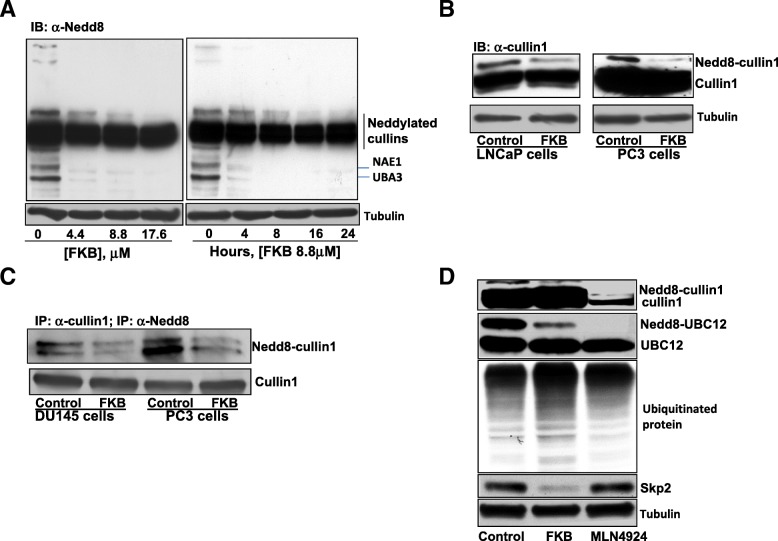
FKB inhibits the neddylation of Cullin1 and Ubc12. **a**, PC3 cells were treated with indicated concentrations of FKB for different periods of time. Expression of NEDD8 and its modified proteins were measured by Western blotting analysis. **b**, Western blotting analysis of Cullin1 neddylation in vehicle control (0.1% DMSO) or 8.8 μM FKB treated LNCaP and PC3 cells. **c**, Cullin1 neddylation was measured via immunoprecipitation with anti-Cullin1 and then Western blotting analysis of the immunoprecipitates by anti-NEDD8 antibody in vehicle control or 8.8 μM FKB treated DU145 and PC3 cells. **d**, PC3 cells were treated with 8.8 μM FKB or 1 μM MLN4924 for 16 h. Neddylation of Cullin1 and Ubc12 and expression of ubiquitinated proteins and Skp2 were examined by specific antibodies and β-tubulin serves as a loading control

We have identified that FKB is the most potent apoptosis inducer of chalcones isolated from kava root extracts for inhibition of the growth of prostate cancer cell lines with minimal effects on normal prostate epithelial cells [13]. A further screening assay suggested that FKB may function as a neddylation inhibitor (data not shown). We therefore examined the expression of NEDD8 and its modified proteins in PC3 cells after FKB treatment. FKB treatment decreased neddylation of multiple proteins, including Cullins, NAE1, UBA3, etc. in both a dose- and time- dependent manner (Fig. 1a). Western blotting analysis confirmed that FKB reduced Cullin-1 neddylation in LNCaP and PC3 cells (Fig. 1b). In addition, immunoprecipitation experiments were conducted by using anti-Cullin-1 antibody to pull down its associated complexes and detecting the pull down Cullin-1 with an anti-NEDD8 antibody. The result demonstrates that the level of NEDD8-modified Cullin-1 decreases in both DU145 and PC3 cells when treated with FKB (Fig. 1c). In addition, we used a reported neddylation inhibitor, MLN4924, as a positive control for deneddylation [6–10]. Though the effect of FKB on de-neddylation was weaker than MLN4924, FKB treatment resulted in an increased ubiquitination of proteins accompanied by Skp2 down-regulation, whereas MLN4924 neither increase the expression of ubiquitinated proteins nor decrease Skp2 expression (Fig. 1d). This result suggests that FKB is a novel neddylation inhibitor with a different mechanism from the known neddylation inhibitor MLN4924.

### FKB interacts with NAE1 regulatory subunit to inhibit UBC12 neddylation

**Fig. 2:**
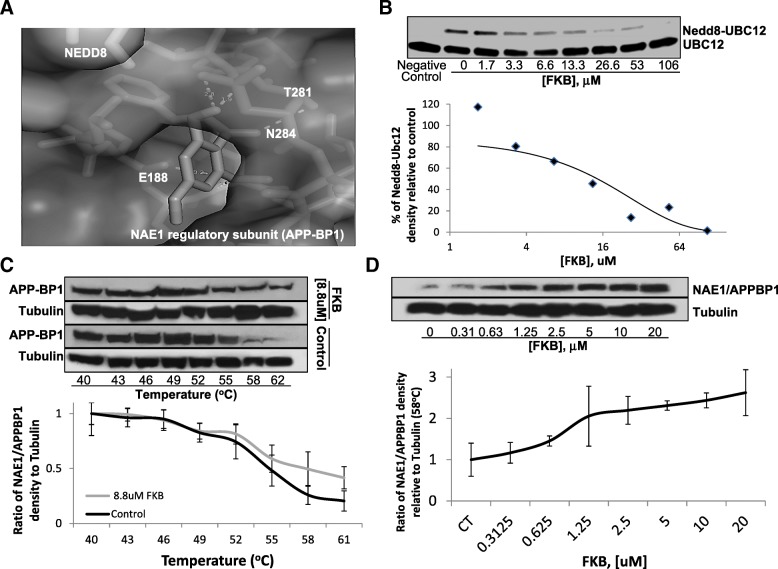
FKB interacts with the NAE1 to inhibit Ubc12 neddylaton. **a**, Autodocktools program was used to dock FKB with the NAE1 protein (3gzn), which has a predicted IC_50_ of 986.1 nM, and Pymol program was used to observe the superimposed binding of FKB to NAE1. FKB was predicted to bind within the E1 regulatory subunit (grey) of NAE1. **b**, In vitro Ubc12 neddylation initiation assay was performed. Ubc12 antibody was used to detect the changes of NEDD8- conjugated Ubc12 bands, which are inhibited by FKB treatments. Densitometry analysis of Western blotting bands shows an estimated IC_50_ of ~ 11 μM for FKB to inhibit Ubc12 neddylation. **c**, Cellular thermal shift assay was performed using PC3 cells treated with 8.8 μM FKB or 0.1% DMSO under a range of temperatures from 40^c^C to 62 °C. Western blotting bands were semi-quantified by densitometry analysis and adjusted by loading control β-tubulin. The line graph shows relative changes of density ratios from 40^c^C to 62 °C. Error bars represent standard deviations of three replicates. **d**. Cellular isothermal dose response was examined on PC3 cells at 58 °C and treated with FKB at concentrations ranged from 0.3125 μM to 20 μM, where 0.1% DMSO was used as a vehicle control. The line graph shows relative changes of density ratios from different concentrations of FKB treatment relative to vehicle control. CT denotes control. Error bars represent standard deviations of three replicates

Computational model was used to determine the potential binding sites of FKB to NAE1, Ubc12, and Cullin1. NAE1 had the highest predicted inhibition constant of approximately 986 nM by FKB (Fig. 2a), while best predicted inhibitory constant for Ubc12 and Cullin1 were 6.56 μM and 7.07 μM of FKB (Data not shown), respectively. In order to verify the direct effect of FKB on neddylation, an in vitro neddylation initiation experiment was performed where FKB was added to the reaction system of neddylation enzymes in a dose-dependent reaction. The inhibition of Ubc12 neddylation was shown by non-denaturing Western blotting analysis, and an increasing dose of FKB was able to inhibit neddylation to Ubc12 (Fig. 2b, top). The relative density of NEDD8-Ubc12 to the control was analyzed to have an estimated IC_50_ of approximately11μM (Fig. 2b, bottom). These results suggest that FKB inhibits neddylation by hindering NAE1 activities.

CETSA was also performed to determine the direct interaction between FKB and NAE1. Of the NAE1 complex, the regulatory subunit, APP-BP1, was found to be significantly shielded from degradation at an optimal temperature of 58 °C (Fig. 2c) when co-incubated with FKB at the optimal peak concentration of 8 .8μM (Fig. 2d), strongly suggesting that FKB directly binds to the regulatory subunit.

### FKB accelerates Skp2 degradation

**Fig. 3:**
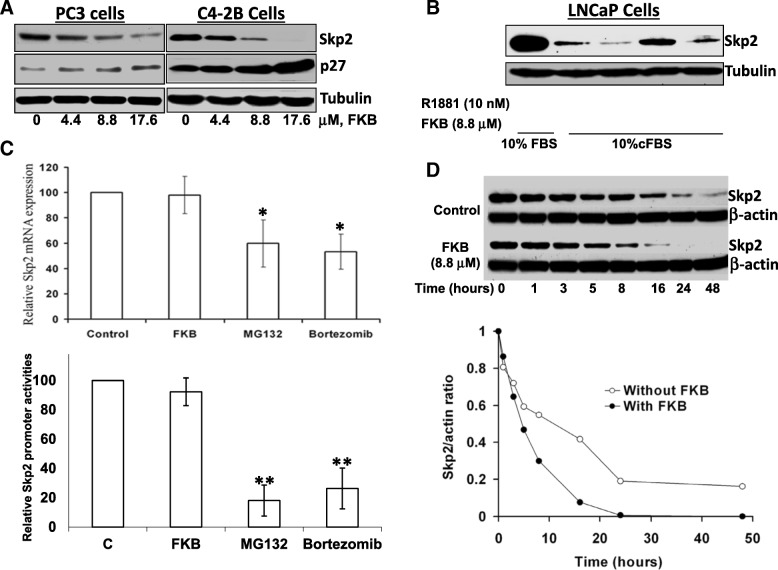
FKB downregulates Skp2 expression via protein degradation. **a**, Western blotting analysis of protein expression of Skp2 and p27/Kip1 in PC3 and C4-2B cells that were treated with vehicle control (0.1% DMSO) or indicated concentrations of FKB for 24 h. β-tubulin was used as a loading control. **b**, LNCaP cells were cultured under 10% charcoal stripped FBS with or without 10 nM synthetic androgen R1881 and then treated with vehicle control (0.1% DMSO) or 8.8 μM FKB for 24 h. The protein expression of Skp2 was decreased by FKB treatment. **c**, PC3 cells were treated with 0.5% DMSO, 8.8 μM FKB, 5 μM MG132 or 10 nM Bortezomib for 16 h. Real-time RT-PCR was performed to analyze mRNA expression of Skp2. PC3 cells were co-transfected with Skp2-Luc along with a Renilla luciferase plasmid pGL 4.71 and Luciferase activities were measured. Proteasome inhibitors MG132 and Bortezomib but not FKB significantly decrease mRNA expression and promoter activity of Skp2 (Student t test, Ps < 0.05). Bars are mean ± SD of three independent experiments. **d**, PC3 cells were treated with 10 μg/L of cycloheximide (CHX). After 16 h of treatment, the cells were treated in absence or presence of 8.8 μM of FKB. Western blotting was performed to determine Skp2 protein levels and quantified by densitometry with Image J software and adjusted for loading control. FKB reduces Skp2 protein stabilities over time

Next, we have examined the effect of FKA on the down-stream events of the neddylation pathway, such as expression of an E3 ligase. FKB was shown to have a dramatically downregulating effect on Skp2 protein levels at a concentration of 8.8 μM in both androgen receptor-positive C4-2B and androgen receptor negative PC3 cells (Fig. 3a). Subsequently, the protein expression of p27/Kip1, the major target substrate of Skp2, was also shown to be increased (Fig. 3a). We then determine the effects of FKB on Skp2 expression under androgen-stimulated and androgen-deprived conditions. Androgen deprivation reduces the protein expression of Skp2 and addition of synthetic androgen into androgen deprived media restores the expression of Skp2 in LNCaP cells. When we treated LNCaP cells in presence or absence of androgen and with FKB together, we found Skp2 was decreased by FKB, regardless of if there were a presence or absence of androgen (Fig. 3b). We next determined whether FKB can affect Skp2 mRNA expression levels and Skp2 promoter activities. Unlike proteasome inhibitors MG132 and Bortezomib, we found that FKB had no significant effect on either Skp2 mRNA expression or the transcriptional activity of the Skp2 promoter (Fig. 3c). These results indicate that the decrease of Skp2 was likely due to the reduced protein stability of Skp2 protein. We therefore employed cycloheximide to block de novo protein synthesis and recorded the degradation rate of Skp2 in a time course treatment of PC3 cells. We analyzed the Skp2 protein expression levels over time and found that the rate of Skp2 degradation is significantly increased under FKB treatment (Fig. 3d).

### FKB increases Skp2 ubiquitination leading to its degradation

**Fig. 4:**
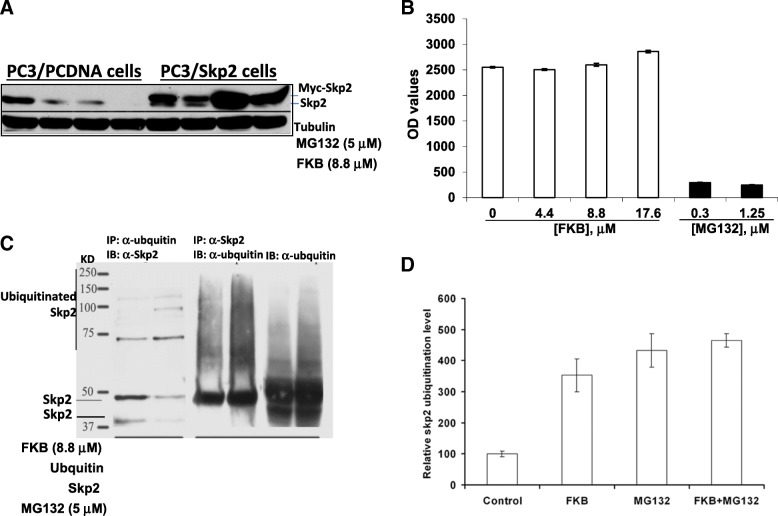
FKB degrades Skp2 protein in a proteasome and ubiquitination dependent manner. **a**, Western blotting analysis of Myc-Skp2 and endogenous Skp2 protein expression after PC3/Skp2 or PC3/PCDNA cells were treated with FKB, MG132 or their combination for 24 h. **b**, proteasome activities (i.e. OD values) were measured by the 20S Proteasome Assay Kit after PC3 cells were treated with 0.1% DMSO, FKB or MG132 for 24 h. **c**, Skp2 ubiquitination was measured by immunoprecipitation with anti-ubiquitin antibody and then Western blotting analysis of immunoprecipitates by anti-Skp2 after PC3 cells were treated with 0.1% DMSO or FKB (left two panels). 293 cells were transiently transfected with Skp2 and ubiquitin expression plasmids. After 24 h of transfection, cells were treated with 0.1% DMSO or FKB for 16 h. FKB increases ubiquitination of Skp2. **d**, the relative ubiquitination levels were measured by the Ubiquitylation Assay Kit after DU145 cells were treated by 0.1% DMSO, 8.8 μM FKB, 5 μM MG132 for 16 h

To evaluate whether the Skp2 degradation by FKB depends on the proteasome function, PC3 cells with or without ectopic expression of Skp2 were treated with FKB, proteasome inhibitor MG132 alone or their combination. Figure 4a shows that FKB and MG132 combination resulted in enhanced down-regulation of endogenous Skp2 compared to either alone, whereas the degradation of ectopically expressed Skp2 protein (which is driven by the CMV promoter but not endogenous transcriptional factors) by FKB was completely blocked by MG132. This result indicates that the FKB induced Skp2 degradation relies on intact proteasome function. Figure 4b have further demonstrated that FKB has no effect on the proteasome function at its concentrations for inducing Skp2 degradation when compared to MG132. In addition, we have observed that the ubiquitinated Skp2 level was increased under FKB treatment in both Skp2 and ubiquitin immunoprecipitation assays (Fig. 4c left and middle panel), and the whole ubiquitination level in cell lysate was also increased marginally (Fig. 4c right panel). A quantitative analysis of ubiquitination level of Skp2 also demonstrated a significant increase in ubiquitin modified Skp2 when treated with FKB (Fig. 4d). Together, these results demonstrate that FKB decreases Skp2 by increasing its ubiquitination, which can be recognized and degraded by proteasome and its degradation in the proteasome without affecting the proteasome function itself.

### FKB induced Skp2 degradation is dependent on a functional Cullin-1

**Fig. 5:**
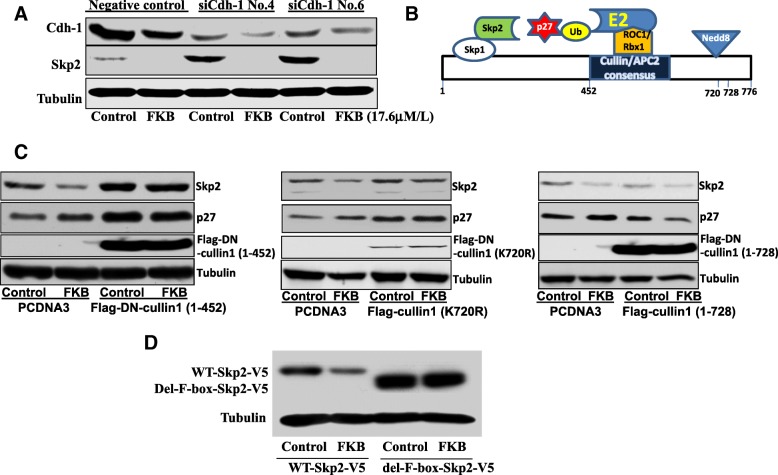
Degradation of Skp2 by FKB is dependent on Cullin1 activity. **a**, PC3 cells were transiently transfected with siRNA control or Cdh1siRNAs. The knockdown effects on Skp2 expression were evaluated by Western blotting analysis. FKB decreases Skp2 expression under Cdh1 knockdown conditions. **b**, cartoon depiction of ubiquitin transfer from E2 to E3 substrate in the Cul1–Rbx1–Skp1–F box^Skp2^ SCF ubiquitin ligase complex. **c,** left panel: PC3 cells stably expressing dominant-negative (DN) Cullin1 or vector control (PcDNA3), middle panel: 293 T cells were transiently transfected with mutant Cullin-1 (K720R) or PcDNA3, and right panel: 293 T cells were transiently transfected with full-length Cullin1 (1–728). Cells were treated with 0.1%DMSO or 8.8 μM FKB for 24 h. Skp2 and p27/Kip1 expression was examined by Western blotting analysis. **d**, PC3 cells were transfected with F-box deleted Skp2 (V5-del-F-box-Skp2). After 24 h of transfections, cells were treated with 0.1%DMSO or 8.8 μM FKB for 24 h, and Skp2/V5 expression was examined by Western blotting analysis

There are at least two reported mechanisms involved in Skp2 ubiquitination and degradation [19]. One is that Skp2 is ubiquitinated by another E3 ligase complex, such as the APC/C complex with Cdh1, the other one being the Skp1-Cullin-1-ROC/Rbx1 complex which is referred to as self-ubiquitination of Skp2. We first knocked down Cdh1 by siRNA and found that FKB-induced Skp2 decrease is not restored in the Cdh1 knock down conditions and that Cdh1 knockdown led to increased protein expression of Skp2 (Fig. 5a). This result suggests that FKB induced Skp2 degradation is not dependent on the expression of Cdh1.

We therefore turned our investigation into the Cullin-1, which is post-transcriptionally modified by neddylation and serve as a bridge to link ROC/Rbx1/ ubiquitin-conjugating enzyme E2 to the Skp1/Skp2 complex for ubiquitin transfer. A full-length wild-type Cullin1 expression plasmid, a dominant negative Cullin-1 expression plasmid that retains the binding to the Skp1/Skp2 complex but lacks the E2 (i.e. ROC/Rbx1) binding domain, or a mutant Cullin-1(K720R) with disruption of the NEDD8 conjugation site expression plasmid as shown in Fig. 5b was then transfected into 293 T cells. After transfection, these transfected cells were treated with 8.8 μM FKB for 24 h. Western-blot analysis shows that FKB induced Skp2 degradation was fully blocked when Cullin1 activities were hindered by expression of dominant-negative or neddylation site deleted Cullin-1 (Fig. 5c). The expression of F-box-deleted Skp2 protein that losses its binding to the Cullin-1 complex via Skp1 also cannot be decreased by FKB treatment (Fig. 5d). As suggested by these results, a functional Cullin1 that acts as a bridge for transferring ubiquitin from E2 to E3 ligase may be required for FKB induced Skp2 degradation.

Furthermore, NEDD8 is removed from cullins by specific isopeptidase activity of the COP9/signalosome (CSN) complex, including CSN5 [25]. siRNA knockdown of CSN5, Ubc12, or Skp1 in PC3 cells decrease the expression of Skp2 and is not able to rescue the effect of FKB induced Skp2 degradation. These results suggest that FKB induced Skp2 degradation doesn’t require the expression of CSN5, Ubc12 and Skp1 (Additional file 1: Figure S1).

### FKB enhances the anti-cancer effects of proteasome inhibitors via Skp2 down-regulation

We have shown that proteasome inhibitors (MG132 and Bortezomib) inhibit the mRNA expression and promoter activity of Skp2 in PC3 cells, indicating a mechanism through transcriptional down-regulation of Skp2 (Fig. 3c). Consistently, proteasome inhibitors (MG132 and Bortezomib) had more potent effect on reducing cell viabilities of Skp2 overexpressing PC3/Skp2 cells than PC3/PcDNA cells with less Skp2 expression, which suggests that Skp2 is a potential target for the growth inhibitory effect of these proteasome inhibitors (Additional file 1: Figure S2). The IC_50s_ of MG132 and Bortezomib for PC3/Skp2 cells were estimated to be approximately 252.2 ± 34.1nM and 14. 1 ± 1. 7 nM, respectively, compared to 832.7 ± 45.7nM and more than 20 nM for PC3/PcDNA cells (Ps < 0.05).

**Fig. 6:**
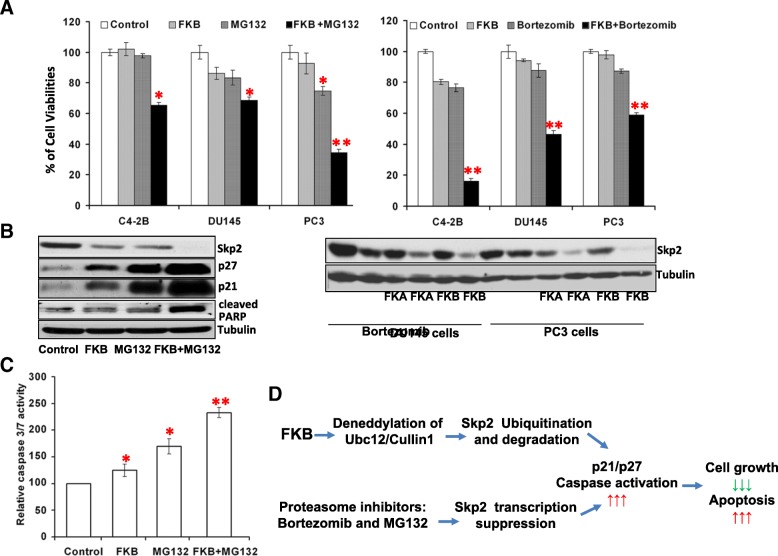
The combined effects of FKB and proteasome inhibitors MG132 and Bortezomib on cell viabilities, apoptosis and expression of Skp2 and p27/Kip1. **a**, C4-2B, DU145 and PC3 cells were treated with 0.1%DMSO, 8.8 μM FKB, 5 μM MG132, 5 nM Bortezomib or their combinations for 24 h. MTT assay was performed to measure cell viabilities. Bars are mean ± SD of three independent experiments. “*” denotes *P* < 0.05 and “**” denotes *P* < 0.01. **b**, left panels: protein expression of Skp2, p27/Kip1, p21/WAF1, and cleaved PARP was measured after DU145 cells were treated with 0.01% DMSO, 8.8 μM FKB, 5 μM MG132), or their combinations for 24 h. Right panels: DU145 and PC3 cells were treated with 8.8 μM FKB, 20 μM flavokawain A (FKA), 5 nM Bortezomib, or their combinations. Skp2 protein levels were examined. **c**, the caspase 3/7 activities were measured by ELISA kit after DU145 cells were treated with 0.1% DMSO, 8.8 μM FKB, 5 μM MG132, and their combination for 24 h. Bars are mean ± SD of three independent experiments. **d**, schematic presentation of mechanisms for the combined effects of FKB and proteasome inhibitors. FKB degrades Skp2 via inhibition of Ubc12/Cullin1 neddylation, whereas proteasome inhibitors down-regulation of Skp2 expression through transcriptional suppression. The combination results in enhanced up-regulation of p21/WAF1, p27/Kip1, and cleaved PARP, leading to greater growth inhibition and apoptosis

Since FKB targets Skp2 degradation for its growth inhibitory effect on prostate cancer cell lines (Fig. 3 and Additional file 1: Figure S3), we examined whether the anti-prostate cancer effects of MG132 or Bortezomib could be enhanced via combination with FKB. Figure 6a shows that MG132, Bortezomib or FKB alone at concentrations minimally to slightly inhibit the growth of prostate cancer cell lines (i.e. C4-2B, DU145 and PC3), whereas their combinations results in approximately 30 to 84% growth inhibition on these cell lines, respectively, suggesting an additive to synergistic effect dependent on cell lines. Western blotting analysis further demonstrates that MG132, Bortezomib or FKB either alone decreases the protein expression of Skp2 by less than 50%, and that their combinations results in a complete inhibition of Skp2 protein expression (Fig. 6b and Additional file 1: Figure S4). Furthermore, combination of FKB with MG132 lead to markedly enhanced expression of cell cycle inhibitors p27/Kip1 and p21/WAF1 (Fig. 6b). Furthermore, combination of FKB and MG132 causes an increased cleavage of PARP and Caspase 3/7 compared to each treatment alone (Fig. 6c), indicating an enhanced apoptosis by the combination.

**Fig. 7:**
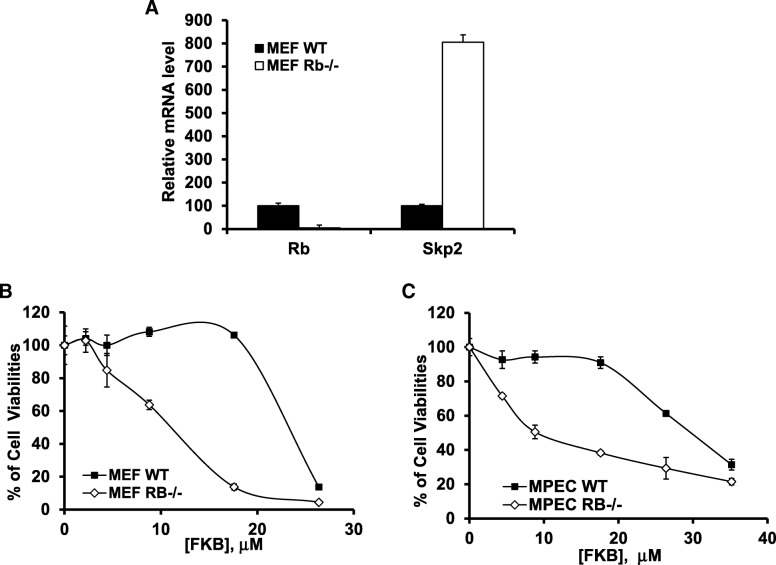
FKB selectively inhibits the growth of RB deficient cells compared to RB wild type cells. **a**, mRNA expression of Rb and Skp2 was analyzed via RT-PCR and qPCR in MEF wild-type and Rb−/− cell lines. **b**, Mouse embryonic fibroblasts (MEFs) and **c,** mouse prostate epithelial cells (MPECs), wild-type and Rb−/−, were treated with 0.1% DMSO or indicated concentration of FKB for 72 h. Cell viabilities were measured via MTT assays

Defects in the RB1 tumour suppressor are one of the more common driver alterations in prostate cancer progression [26–31] and Skp2 was shown to be required for RB1 loss initiated pituitary tumorigenesis [32]. Here, we observed that the mRNA levels of Skp2 in RB1 knockout mouse embryonic fibroblasts (MEFs) are approximately 8 fold higher than those in wild-type MEFs (Fig. 7a). In addition, we demonstrate that FKB selectively inhibits the growth of RB1 deficient cells: The IC_50s_ of RB−/− MEFs and mouse prostate epithelial cells (MPECs) were estimated to be approximately 12 μM and 10 μM compared to IC_50s_ of their wild type control counterparts 25 μM and 31 μM, respectively (Fig. 7b and c, Ps < 0.05).

Our results can be simply summarized as Fig. 6d, which indicates that the combination of suppressing Skp2 transcription by proteasome inhibitors and inducing Skp2 protein degradation via FKB may cause synergistic downregulation Skp2 expression leading to pronounced up-regulation of p27/Kip1 andp21/WAFs, activation of caspase cascade, cell growth inhibition and apoptosis. RB deficient prostate cancer may be particularly sensitive to the FKB and Bortezomib combination therapy.

## Discussion

The results from our experiment support the hypothesis that FKB’s inhibitory effect on prostate cancer cells is due to FKB’s binding to the NAE1 regulatory subunit APP-BP1. Our CETSA results confirm that FKB directly binds to APP-BP1, which in turn was our prediction based on molecular docking. This binding results in two distinct events. The first is that FKB treatment prevents neddylation of Cullin-1 and Ubc12. The second distinct event is that treatment with FKB simultaneously causes a downstream ubiquitination and degradation of E3 ligases SKP2. These observations suggest that FKB is distinct from a known neddylation inhibitor MLN4924, which forms NEDD8-MLN4924 adduct to inhibit neddylation of Cullin-1 and Ubc12 [5–10] but didn’t induce protein ubiquitination and Skp2 degradation.

In addition, FKB demonstrates two characteristics that make FKB or its derivatives valuable for further investigation of its usefulness in combination therapy for prostate cancer. The first one is that FKB selectively inhibits the growth of RB deficient cells by degradation of Skp2. RB-deficient prostate tumors present a significant clinical challenge [26–31]. Loss of RB function was found with high frequency in castration-resistant prostate cancer [26–31]. In one way, most RB defective prostate tumors are associated with high androgen receptor expression, poor prognosis, and resistance to hormone therapy [29, 30]. Clinically, prostate cancer patients with early loss of RB function were often found to be those men whose absolute PSA value does not go down below 0.2 ng/ml after androgen depletion [29, 30]. In the other way, prostate cancer small cell/neuroendocrine phenotype, an increasingly prevalent histologic subtype in castration resistant prostate cancer with low androgen receptor activity is also characterized by loss of RB expression [31]. Our previous studies have demonstrated that FKB is more potent in reducing cell viabilities of androgen receptor negative, castration resistant prostate cancer cell lines [13], and that FKB transcriptionally down-regulates the expression of androgen receptor and its target genes leading to inhibition of in vivo tumor growth in patient derived xenograft models [12]. Skp2 expression has been shown to be required for RB loss initiated pituitary tumorigenesis in mouse models [32]. More recently, multiple components of the SCF^SKP^Cullin F box containing complex including Skp2 has been identified to be candidates of highly penetrant, synthetic lethal interactions in RB defective triple negative breast cancer [33]. Taken together, these results provide a strong rationale that targeting Skp2 by FKB, its derivatives, or proteasome inhibitors (i.e. Bortezomib) should be evaluated as a novel approach for treatment of RB defective, castration resistant prostate cancer.

While FKB is not as potent as MLN4294 for inhibition of neddylation, the second characteristic of FKB is that when combined with the proteasome inhibitors Bortezomib or MG132, the anti-prostate cancer effects were significantly enhanced. The combination of Bortezomib with FKB for enhanced anti-prostate cancer effects is also mechanistically or rationally justified, given that Bortezomib and FKB act through two distinct mechanisms for down-regulation of Skp2 expression: one is through suppression of transcription and the other is through protein ubiquitination and degradation. Furthermore, inhibition of Skp2 has been shown to overcome the resistance to Bortezomib in multiple myeloma [20].

## Conclusion

In conclusion, FKB is a new inhibitor of protein neddylaiton, which is mechanistically distinct from MLN4924 (Pevonedistat), a known neddylation inhibitor currently on clinical trials for treatment of cancers [5–10]. FKB directly interacts with the NAE1 regulatory subunit APP-BP1, resulting in deneddylation of Ubc12 and Cullin1, reduced activity of the SCF^SKP2^ complex and SKP2 ubiquitination and degradation, as well as up-regulation of p21/WAF1 and p27/Kip1and activation of the caspase mediated apoptotic pathway. When combined with Bortezomib, the growth inhibitory effect was increased than either alone, indicating FKB as a promising candidate for drug combination therapy of prostate cancer. Further research will be directed at the synthetic lethal interaction of RB loss with Skp2 overexpression in castration-resistant prostate cancer and targeting of Skp2 by combination of Bortezomib and FKB or its derivatives for treatment of RB deficient, castration resistant prostate cancer.

## Additional file

Additional file 1: Supplmentary **Figures S1-S4**. (PPTX 392 kb)

## Abbreviations

APP-BP1: Amyloid Precursor Protein-binding Protein1
CETSA: cellular thermal shift assay
Cks1b: Cyclin-dependent kinases regulatory subunit 1
Commd1: Copper Metabolism Domain Containing 1
CSN5: COP9/signalosome subunit 5
DCN1: Defective in cullin neddylation protein 1
DMSO: Dimethyl sulfoxide
FKB: Flavokawain B
MEFs: Mouse embryonic fibroblasts
MPECs: mouse prostate epithelial cells
NAE: NEDD8 activating enzyme
NEDD8: Neural Precursor Cell Expressed, Developmentally Down-Regulated 8
ROC/Rbx1: regulator of cullins-1/RING box protein1
SCF: the Skp1-Cullin-F box protein complex
siRNA: small interfering Ribonucleic Acid
UBA3: Ubiquitin Like Modifier Activating Enzyme 3
UBE2M/Ubc12: NEDD8-conjugating enzyme 2 M
