# Supplementary material for: Flavokawain B targets protein neddylation for enhancing the anti-prostate cancer effect of Bortezomib via Skp2 degradation
Source: Cell Commun Signal. 2019 Mar 18;17:25. doi: 10.1186/s12964-019-0338-2 (PMC6423783; doi:10.1186/s12964-019-0338-2)
Supplement: Supplementary file 1 — Supplmentary Figures S1-S4. (PPTX 392 kb) [file 12964_2019_338_MOESM1_ESM.pptx]

## Slide 1
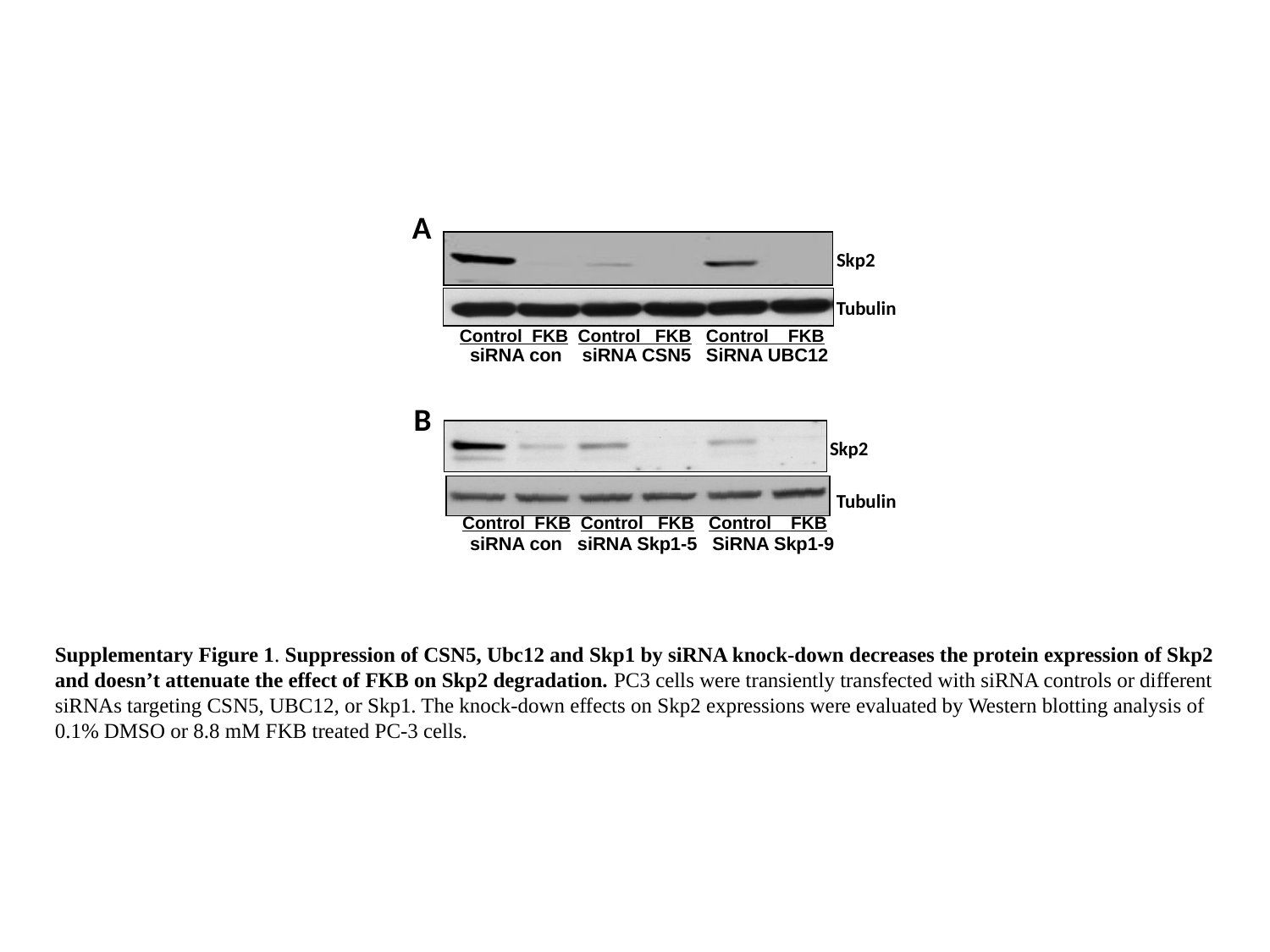

A
Skp2
Tubulin
Control FKB Control FKB Control FKB
siRNA con siRNA CSN5 SiRNA UBC12
B
Skp2
Tubulin
Control FKB Control FKB Control FKB
siRNA con siRNA Skp1-5 SiRNA Skp1-9
Supplementary Figure 1. Suppression of CSN5, Ubc12 and Skp1 by siRNA knock-down decreases the protein expression of Skp2 and doesn’t attenuate the effect of FKB on Skp2 degradation. PC3 cells were transiently transfected with siRNA controls or different siRNAs targeting CSN5, UBC12, or Skp1. The knock-down effects on Skp2 expressions were evaluated by Western blotting analysis of 0.1% DMSO or 8.8 mM FKB treated PC-3 cells.

## Slide 2
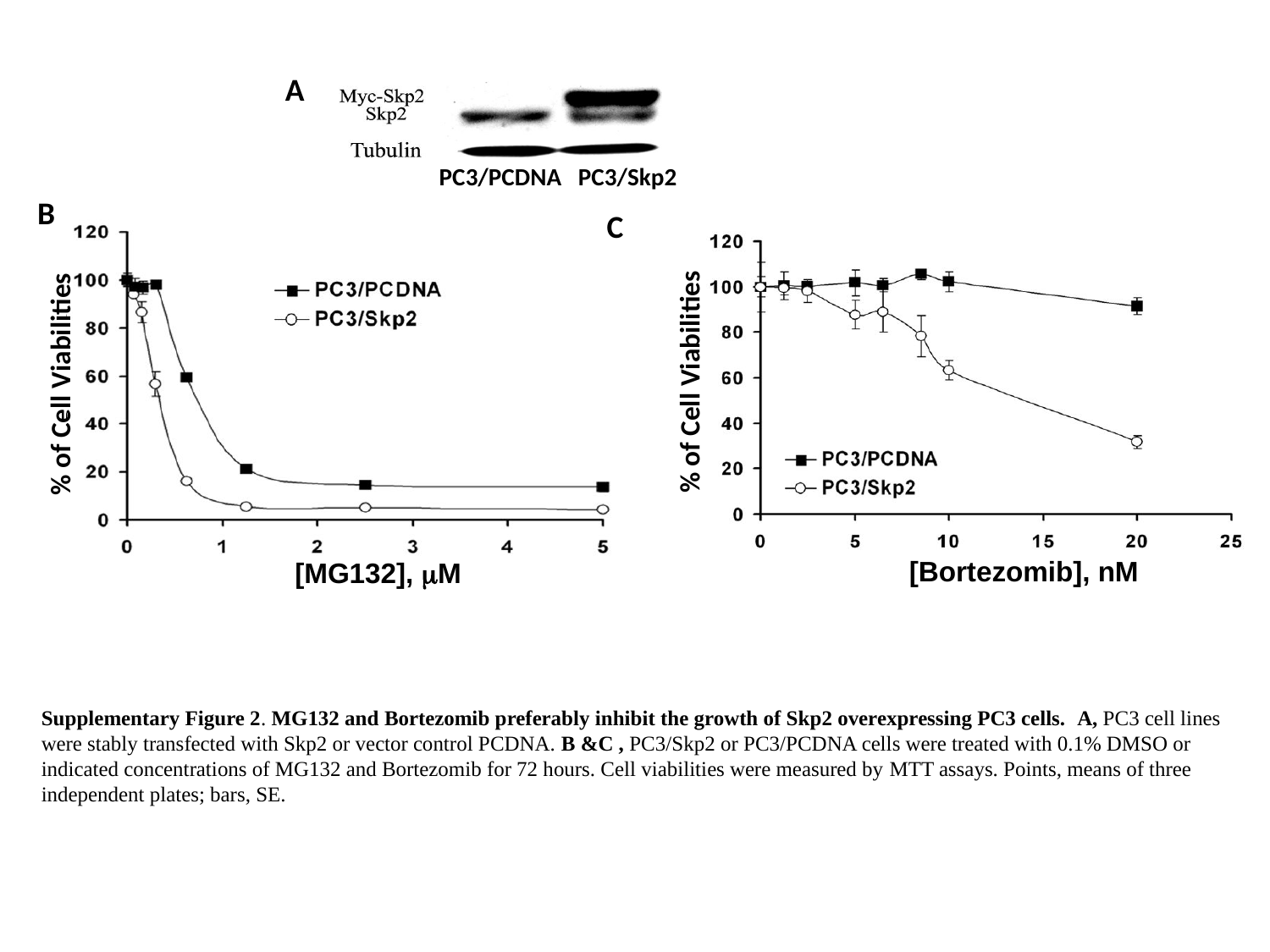

A
PC3/PCDNA PC3/Skp2
B
C
% of Cell Viabilities
[MG132], mM
% of Cell Viabilities
[Bortezomib], nM
Supplementary Figure 2. MG132 and Bortezomib preferably inhibit the growth of Skp2 overexpressing PC3 cells. A, PC3 cell lines were stably transfected with Skp2 or vector control PCDNA. B &C , PC3/Skp2 or PC3/PCDNA cells were treated with 0.1% DMSO or indicated concentrations of MG132 and Bortezomib for 72 hours. Cell viabilities were measured by MTT assays. Points, means of three independent plates; bars, SE.

## Slide 3
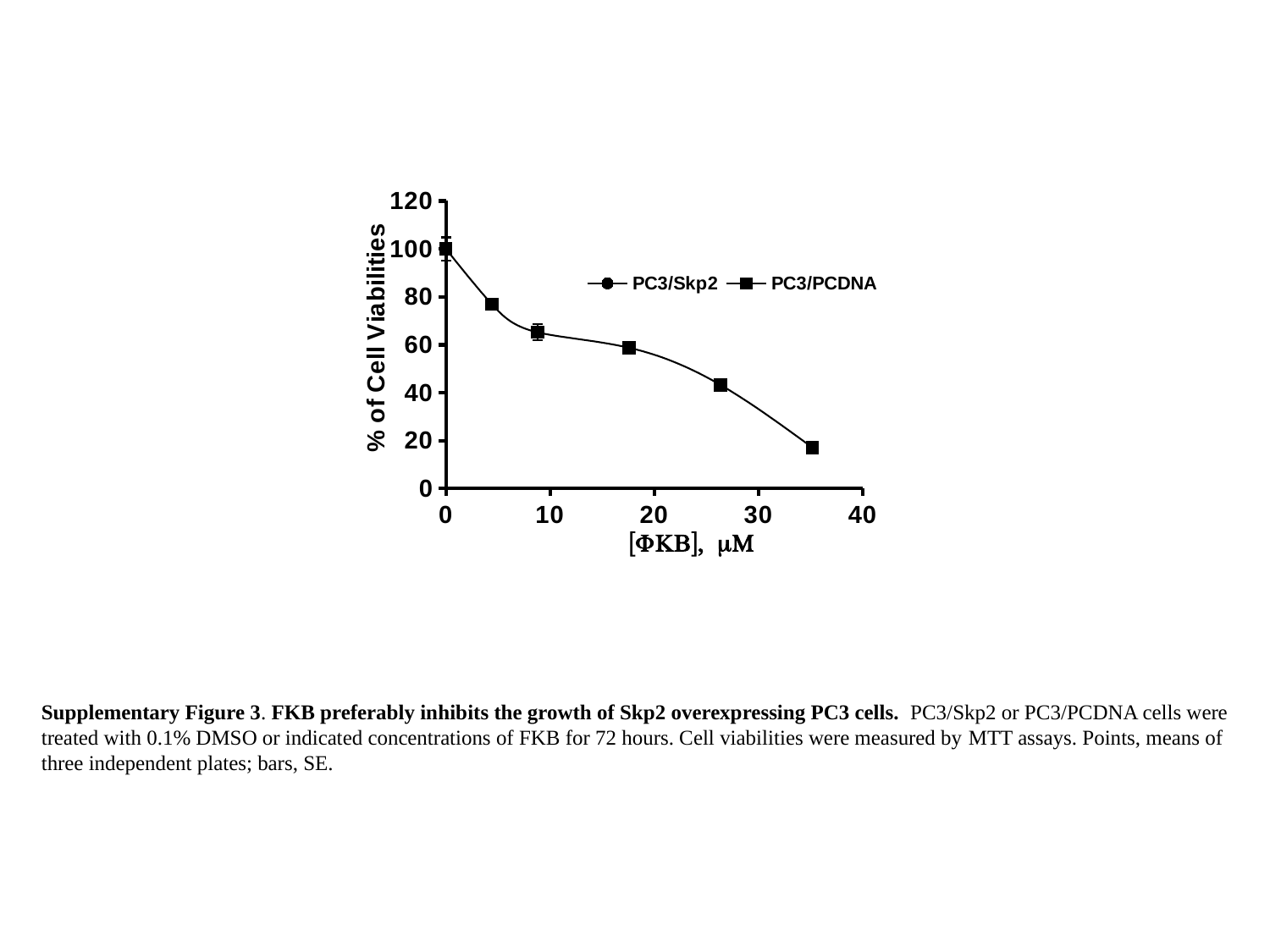

### Chart
| Category | | |
|---|---|---|Supplementary Figure 3. FKB preferably inhibits the growth of Skp2 overexpressing PC3 cells. PC3/Skp2 or PC3/PCDNA cells were treated with 0.1% DMSO or indicated concentrations of FKB for 72 hours. Cell viabilities were measured by MTT assays. Points, means of three independent plates; bars, SE.

## Slide 4
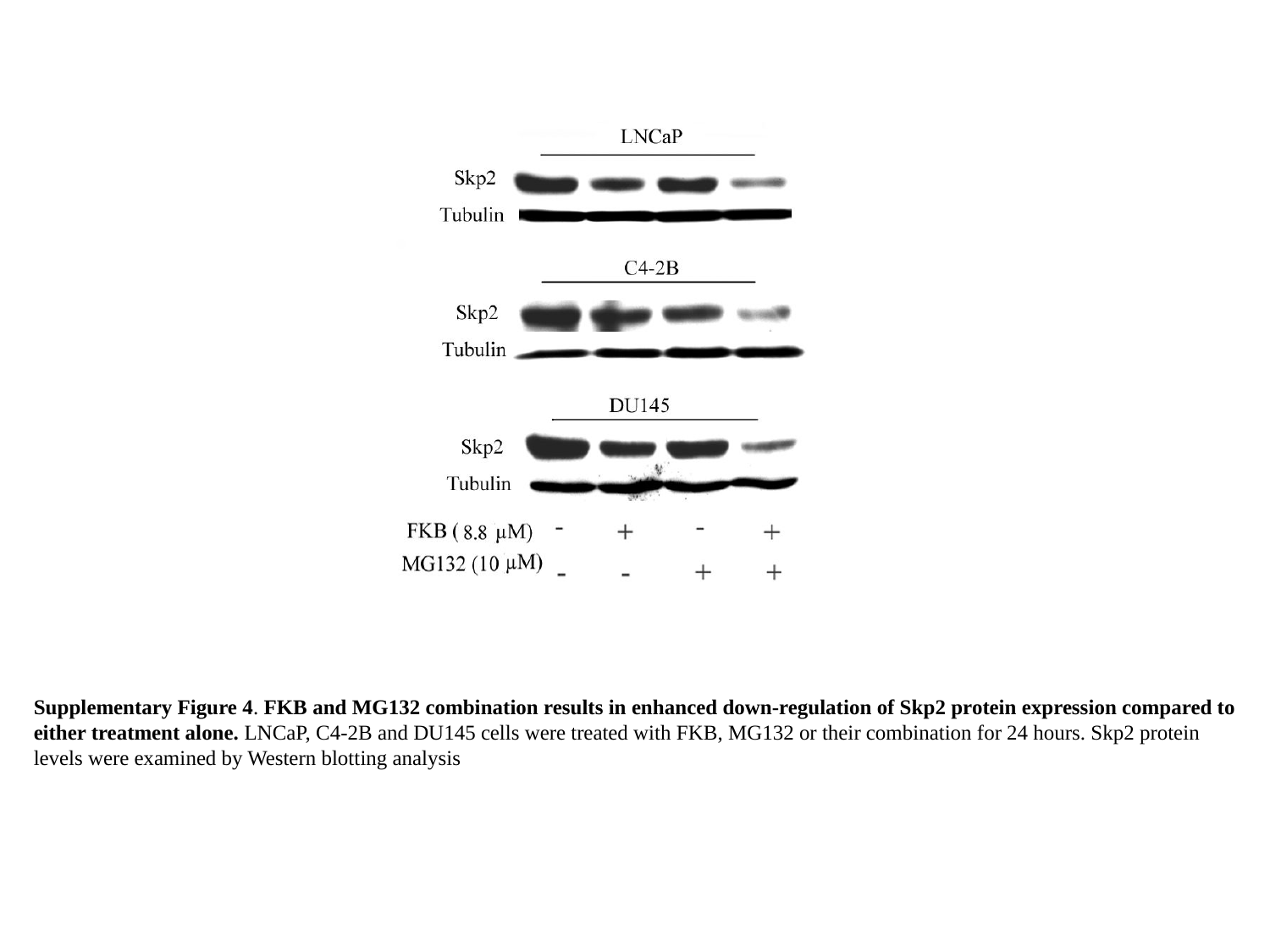

Supplementary Figure 4. FKB and MG132 combination results in enhanced down-regulation of Skp2 protein expression compared to either treatment alone. LNCaP, C4-2B and DU145 cells were treated with FKB, MG132 or their combination for 24 hours. Skp2 protein levels were examined by Western blotting analysis
